# Highlight report: Limits of prognostication of non-small cell lung cancer

**DOI:** 10.17179/excli2017-508

**Published:** 2017-05-31

**Authors:** Alshaimaa Adawy

**Affiliations:** 1 Medical Biochemistry Department, Faculty of Medicine, Alexandria University, Alexandria, Egypt

## ⁯

Recently, Patrick Micke and colleagues from Uppsala University have contributed an outstanding publication on the limitations to predict prognosis in non-small cell lung cancer (Grinberg et al., 2017[6]). For this purpose the authors used two cohorts of 354 and 357 patients, respectively, that were analyzed by immunostaining. Moreover, genome wide expression data of 1779 non-small cell lung cancer patients were included. Based on expression data and results from previous studies (Therneau, 2015[14]; Barlési et al., 2005[1]; Behrens et al., 2013[2]; Shiba et al., 2000[12]; Younes et al., 1997[15]) five proteins (MKI67, EZH2, SLC2A1, CADM1, and NKX2-1) were chosen for immunohistochemical analyses. As expected, each of the individual five selected proteins was significantly or borderline significantly associated with prognosis. Next, the authors studied the association of the combined five protein factors to clinicopathological data. Interestingly, the model based on protein expression alone did not outperform the model based only on the clinicopathological parameters (Grinberg et al., 2017[6]). Combining protein expression with clinicopathological data did not lead to a significantly improved accuracy of survival prediction.

The authors discuss several possible reasons for the negative result, one of them that global gene expression profiles may have performed better. However, despite the availability of several non-small cell lung cancer cohorts with genome-wide data an improvement over clinicopathological parameters including performance status has not yet been demonstrated. A possible reason not discussed by the authors is that tumor tissue used for biomarker or genome-wide expression analysis was taken by surgery soon after diagnosis, while metastasis occurs usually years later. Eventually, biomarker expression undergoes changes during this period and the surgically obtained tumor tissue may no longer be sufficiently representative of the tumor that finally progresses and leads to death.

Currently, numerous studies are performed in several tumor entities aimed to predict prognosis (Selinski et al., 2017[11]; Hellwig et al., 2016[8]; Djureinovic et al., 2016[4]; Lohr et al., 2015[10]; Ghallab, 2015[5]; Cadenas et al., 2014[3]; Lesjak et al., 2014[9]; Suda and Mitsudomi, 2015[13]; Hammad et al., 2016[7]). Of course, the result of Grinberg and colleagues is quite pessimistic for this field of research on molecular biomarkers for prognostication. It will be interesting, whether similar pessimistic results will be obtained also for other tumor entities in future or whether the limited value of protein biomarkers remains specific for non-small cell lung cancer. 
